# Synthesis and Catalytic Performance of Mo_2_C/MoS_2_ Composite Heterojunction Catalysts

**DOI:** 10.3390/ma17102355

**Published:** 2024-05-15

**Authors:** Congyi Zhang, Zhigang Pan, Yaqiu Tao

**Affiliations:** 1 College of Materials Science and Engineering, Nanjing Tech University, Nanjing 211800, China; 202161203161@njtech.edu.cn (C.Z.); panzhigang@njtech.edu.cn (Z.P.); 2 State Key Laboratory of Materials-Oriented Chemical Engineering, Nanjing 211800, China

**Keywords:** Mo_2_C, MoS_2_, hetrostructure, HER

## Abstract

Hydrogen, as a clean, safe, and efficient energy carrier, is one of the hot energy sources that have attracted much attention. Mo_2_C, due to the introduction of C atoms, makes the atomic spacing of the Mo lattice decrease and changes the width of the d-band, which makes the electronic properties of Mo_2_C similar to that of Pt noble metals, exhibiting excellent electrochemical hydrogen precipitation performance. MoS_2_, due to its special crystal structure and tunable electronic structure, has been widely studied. In this paper, Mo_2_C nanoparticles were prepared by high-temperature carbonization, and then two-dimensional layered MoS_2_ were be loaded on Mo_2_C nanoparticles by the hydrothermal method to synthesize Mo_2_C/MoS_2_ composite catalysts. Their electrochemical hydrogen precipitation (HER) performance under acidic conditions was tested. The above catalysts were also characterized by modern material testing methods such as XRD, SEM, TEM, and XPS. The results showed that the composite catalysts exhibited the most excellent electrochemical hydrogen precipitation performance at Mo_2_C/MoS_2_-3, with the lowest overpotential at a current density of 10 mA cm^−2^, Tafel slope, and electrochemical impedance. At the same time, the electrochemically active area was dramatically enhanced, with good stability under prolonged testing. The catalytic activity was significantly improved compared with that of Mo_2_C and MoS_2_. The characterization and experimental results indicate that the heterogeneous structure of Mo_2_C and MoS_2_ formed a built-in electric field between the two, which accelerated the electron transfer efficiency and provided more active sites. The Mo_2_C/MoS_2_ composite catalyst is a low-cost, easy-to-prepare, and high-efficiency electrochemical hydrogen precipitation catalyst, providing a new idea for developing green and clean energy.

## 1. Introduction

With the continuous development of society, humanity’s demand for energy continues to expand, and the excessive use of traditional fossil fuels has led to environmental problems and the emergence of the crisis of energy scarcity. Here, there is the search for a new type of clean energy to eliminate the dependence on the limited fossil fuels for the sustainable development of humanity, where social stability and progress of the problems faced have an urgent need to be resolved [1].

Hydrogen has emerged as a paragon of clean energy carriers, embodying a prodigious potential for high energy yield per mass (142 MJ kg^−1^), dwarfing even the venerable fossil fuels, bolstering its prominence at the pinnacles of energy research. The gamut of prevalent hydrogen fabrication techniques encompasses photocatalytic [2,3] and electrolytic hydrogen generation [4,5,6], biomass enzymatic degradation [7,8], and conventional fossil-fuel-based hydrogen extraction [9,10]. For example, Marjeta et al. [11] synthesized efficient photocatalysts by epitaxial growth of SrTiO_3_ on Bi_4_Ti_3_O_12_ substrates. The multilayer composite catalysts CoS_2_/MoS_2_ and Bi_2_S_2_/MoS_2_ prepared by Yang et al. [12] exhibit high catalytic activity with high specific surface area and unique surface morphology. Of these modalities, the electrolytic conjuration of hydrogen is lauded for its impeccability and environmental benevolence, juxtaposed with the offscourings of its counterparts.

Electrochemical hydrogen evolution is a process whereby hydrogen gas is produced through the directional charge transfer in an electrochemical reaction. Within the electrode system, cations in the electrolyte migrate towards the cathode, absorbing electrons and undergoing a reduction reaction to generate hydrogen gas; anions in the electrolyte move towards the anode, release electrons, and undergo an oxidation reaction to generate oxygen gas. This method boasts advantages such as efficiency, environmental friendliness, and safety. In electrochemical hydrogen evolution, the electrodes are key in catalyzing the reaction, commonly utilizing transition metal compounds such as platinum, palladium, iron, and nickel, which demonstrate excellent catalytic activity and can significantly reduce the potential required for the electrochemical hydrogen evolution reaction. Molybdenum-based compounds are also among the hot materials currently under research as catalysts for electrochemical hydrogen evolution. The electrolyte solution is typically acidic (0.5 mol L^−1^ H_2_SO_4_) or alkaline (1 mol L^−1^ KOH solution).

Water electrolysis hydrogen evolution is divided into the hydrogen evolution reaction (HER) and the oxygen evolution reaction (OER) [13], each occurring at the cathode and anode of the electrode, respectively. HER is one of the simplest electrochemical reactions, involving a two-electron transfer; OER, on the other hand, involves a four-electron transfer. Compared to OER, HER occurs more easily and is more efficient, with the required overpotential being lower. Therefore, water electrolysis hydrogen evolution is a simple and efficient hydrogen production strategy. The reaction equation for HER is

(1) $$2H^{+}+2e^{-}\to H_{2}$$

This reaction consists of three steps: the Volmer, Tafel, and Heyrovsky reactions [14,15,16], which correspond to different stages in the electrochemical hydrogen evolution process. The Volmer reaction is considered the initial step of the electrochemical hydrogen evolution reaction and is the only step considered in the theoretical model of the single-electron HER. In the Volmer reaction, H^+^ ions adsorb onto the catalyst surface to form adsorbed hydrogen atoms (H*), where * indicates an active site on the catalyst. The Tafel reaction is where two adsorbed H* atoms combine and desorb to form H_2_. The Heyrovsky reaction involves an adsorbed H* combining an H^+^ ion from the electrolyte and an electron e^−^ to desorb and form H_2_. In alkaline conditions, the required H* is produced through the dissociation of H_2_O molecules, and hence the reaction equation varies.


(2) $$\begin{array}{ccc} \;\;\;\;\;\;\;\;\;\;\;\mathrm{Volmer} & & \;\;\;\;\;\;H^{+}+e^{-}\to H^{*}\;(\mathrm{acid}) \end{array}$$

(3) $$\begin{array}{ccc} \;\;\;\;\;\;\;\;\;\;\;\;\mathrm{Volmer} & & \;\;H_{2}O+e^{-}\to H^{*}+OH^{-}\;(\mathrm{alkaline}) \end{array}$$

(4) $$\begin{array}{ccc} \;\;\;\;\;\;\;\;\;\;\;\mathrm{Heyrovsky} & & \;\;\;H^{*}+H^{+}+e^{-}\to H_{2}\;(\mathrm{acid}) \end{array}$$

(5)           Heyrovsky       H2O+H*+e−→H2+OH− (alkaline)

(6) $$\begin{array}{ccc} \;\;\;\;\;\;\;\;\;\;\mathrm{Tafel} & & \;\;\;H^{*}+H^{*}\to H_{2} \end{array}$$

In HER, the Tafel slope is usually used to determine the mechanism of cathodic H_2_ generation: since the HER reaction necessarily includes the process of electron transfer and desorption, when the Tafel slope is around 120 mV dec^−1^, the decisive step of HER is the Volmer reaction, and the electron transfer rate is slow, so the reaction rate can be accelerated by increasing the active sites of the catalyst at this time. When the Tafel slope is around 40 mV dec^−1^, the decisive step of HER is the Heyrovsky reaction, the desorption process is slow, and the reaction rate can be accelerated by changing the physical properties of the catalyst (specific surface area, roughness, etc.). When the Tafel slope is around 30 mV dec^−1^, the electron transfer and desorption rate on the electrode surface are very fast, and then the reaction decisive step is the compound desorption process of H*, i.e., the Tafel reaction, which can be accelerated by increasing the active area of the catalyst as well.

Platinum on carbon (Pt/C) has historically reigned as the superlative catalyst for electrochemical hydrogen production [17], acclaimed for its exceptional efficacy. However, the prohibitive cost of platinum, coupled with its scant availability and vulnerability to catalyst poisoning, invites the imperative of conceiving economical, facilely synthesized, robust, safe, and innocuous catalytic surrogates. Amidst this pursuit, an array of transition metal derivatives, including oxides, carbides, and sulfides, have emerged as contenders exhibiting laudable electrochemical propensities. Liu et al. [18] doped Ru single atoms into CoP nanoparticles and then loaded this catalyst on carbon dots (CDs) to form Ru-CoP/CDs composites. The CDs, which were made from citric acid and ethylenediamine, could regulate the electrocatalytic reaction sites and electronic structure of Ru-CoP/CDs by inhibiting the growth and agglomeration of Ru-CoP, resulting in excellent electrocatalytic performance of the catalysts. In 2021, Park et al. [19] improved the catalytic activity by introducing Ni to form a Ni-Mo binary alloy nitride (Ni_2_Mo_3_N) to weaken the Mo-H bonding and thus improve the catalytic activity, which enabled the catalyst to exhibit excellent catalytic performance. Notably, molybdenum carbide (Mo_2_C) has garnered significant interest as a material of meritorious distinction among electrocatalysts.

Molybdenum carbide [20], a venerated constituent within the pantheon of transition metal carbides, manifests an engorged metallic framework and an augmented interatomic chasm among Mo atoms. This structural transformation mitigates orbital congruency among neighboring metal atoms. Insights from Density Functional Theory (DFT) intimations denote that the electron conjugation across the d orbitals of Mo and the s and p orbitals of carbon engenders a marked augmentation of the d-band in metallic Mo [21]. Such alterations consummate an electronic schema and catalytic comportment mirroring that of the noble metals partaking of Group VIII, thus ensuring a pronounced HER acumen [22].

Utilizing the two-dimensional layered material molybdenum disulfide (MoS_2_), notable for its adjustable electronic properties, robust catalytic stability, and significant natural abundance, MoS_2_ emerges as an exceptional electrocatalyst [23].

Extant literature underscores that monolayer molybdenum disulfide, a consummate archetype of transition metal dichalcogenides (TMDCs), has garnered extensive scrutiny owing to its substantial in-plane electron mobility, pronounced current switching ratio, superior photoelectrocatalytic attributes, and robust catalytic hydrogen evolution activity. Of the trio of molybdenum disulfide allotropes (1T, 2H, and 3R), the 2H phase prevails, owing to its augmented stability, crystallizing into a hexagonal lattice with a pronounced planar stratification. Within the MoS_2_ architecture, Mo atoms are conjoined with S atoms via ionic linkages, whilst S–S interactions are governed by Van der Waals forces, producing a unique sandwich-like configuration that finds application across industries such as aerospace, lubrication, and energy storage. The 1T-MoS_2_, intrinsically metallic, adopts a tetragonal crystalline assembly, and while it is catalytically active, it is inherently unstable, typically transforming to the more stable 2H phase upon annealing. Conversely, the 3R phase of MoS_2_, which crystallizes under high temperatures within the rhombohedral system, is prone to a phase transition into the 2H-MoS_2_ configuration. The preponderance of contemporary research in electrochemical hydrogen evolution orbits around exploring the 2H phase [24,25].

In pursuit of catalytic interfaces conducive to electrochemical hydrogen production—emphasizing potent catalytic efficacy, enduring stability, and economic viability—this investigation involves the synthesis of molybdenum carbide (Mo_2_C) nanoparticles via a high-temperature carbonization route, segueing into the generation of Mo_2_C/MoS_2_ composite heterojunction semiconductor catalysts through the integration of two-dimensional MoS_2_ onto Mo_2_C nanoparticles using hydrothermal methodology. Employing advanced material characterization techniques such as X-ray diffraction (XRD), scanning electron microscopy (SEM), and X-ray photoelectron spectroscopy (XPS), these catalysts were meticulously examined. Their electrochemical hydrogen evolution capability was subsequently evaluated by employing a CHI660E electrochemical workstation. The synthesis revealed that amalgamating Mo_2_C with MoS_2_ considerably ameliorates the electronic structure at both the surface and interface of the catalyst. In the composite, n-type Mo_2_C operates as an electron reservoir, augmenting the electrical conductivity within the layered structure and concurrently unveiling additional active sites. Notably, the hydrogen adsorption-free energy (ΔG_H_) of Mo_2_C is discernibly less negative (−0.65 eV) compared to MoS_2_ (2.05 eV), underscoring the enhanced propensity of Mo_2_C to adhere to H^+^ and conduct the reduction reaction with heightened efficiency [26,27]. The bifurcation of components also culminates in an optimized electrochemical profile for the catalyst. Electrochemical assays ascertain that the composite catalysts outperform isolated Mo_2_C and MoS_2_ nanoparticles in terms of hydrogen evolution. Further optimization of the electronic structure was achieved by fine-tuning the molar ratio of the constituent materials, resulting in an elevation of catalytic performance. In this study, a composite catalyst with a core-shell structure was prepared by innovatively growing MoS_2_ encapsulated on the surface of Mo_2_C grains via a facile synthesis method, which exhibited significantly better electrocatalytic performance than that of Mo_2_C and MoS_2_, as well as showing good stability over a long-time test.

## 2. Materials and Method

### 2.1. Materials

Thiourea (CH_4_N_2_S) was purchased from Tianjin Zhiyuan Chemical Reagent Co. (Tianjin, China). Ammonium molybdate tetrahydrate ((NH_4_)_6_Mo_7_O_24_·4H_2_O, 99%) and 1,8-diaminonaphthalene (C_10_H_10_N_2_) were purchased from Sinophosphate Chemical Reagent Co. (Tianjin, China). Hydrochloric acid (HCl) and sulfuric acid (H_2_SO_4_) were supplied by Nanjing Chemical Specimen Co. (Nanjing, China). All chemicals are analytical grade and used without further purification.

### 2.2. Synthesis of Electrocatalysts

Mo_2_C flakes: In this experiment, pure β-Mo_2_C was prepared by the calcination method. Ammonium molybdate tetrahydrate ((NH_4_)_6_Mo_7_O_24_·4H_2_O) was used as the molybdenum source, and 1,8-diaminonaphthalene (C_10_H_10_N_2_) was used as the carbon source. The calcination precursor was prepared by mixing 2 mmol of ammonium molybdate tetrahydrate in 12 mL of deionized water and 3.2 mmol of 1,8-diaminonaphthalene in 10 mL of anhydrous ethanol. The pH of the mixture was adjusted by 1 mol L^−1^ hydrochloric acid to obtain the precipitation with a molar of 1:2.25 for n (Mo):n (C). The solution was left for 30 min, and then the precipitation was collected and dried at 100 °C for 10 h to obtain the Mo_2_C precursor. The precursor was ground into powder and calcinated in a vacuumed high-temperature tube furnace, which was heated up to 725 °C at a rate of 5 °C min^−1^ and kept warm for 4 h. After that, it was naturally cooled down to room temperature to obtain β-Mo_2_C nanoparticles.

MoS_2_ particles: Ammonium molybdate tetrahydrate was used as the molybdenum source and thiourea (CH_4_N_2_S) as the sulfur source. Typically, 2 mmol ammonium molybdate tetrahydrate and 5.5 mmol thiourea were dissolved in 60 mL of deionized water with stirring to keep a molar ratio of n(Mo): n(S) = 1:4. The black suspension was then transferred into the polytetrafluoroethylene liner of a stainless steel high-temperature hydrothermal reactor. The black suspension was filtered after being heated at 200° for 8 h to obtain the black solid powder, which was washed three times with 95% ethanol and distilled water and then dried in a constant temperature blast oven at 80 °C for 8 h to obtain the MoS_2_ nanoparticles.

Mo_2_C/MoS_2_ composite catalyst: Mo_2_C nanoparticles were prepared using the above method. The composite catalysts with different Mo_2_C/MoS_2_ molar ratios (0.5, 1, 2, 3, and 4) were prepared by the same hydrothermal method using ammonium molybdate tetrahydrate as the molybdenum source and thiourea as the sulfur source, keeping the content of Mo_2_C unchanged.

### 2.3. Characterization

Powder X-ray diffraction patterns were obtained by a Rigaku SmartLab diffractometer (Tokyo, Japan) using Cu Kα (λ = 0.154178 nm) as a radiation source. The morphology of the samples was measured by scanning electron microscope (FESEM, Ultra-55, Zeiss, Oberkochen, Germany). The samples were pretreated at 105 °C for 12 h before measurement. The electrochemical impedance was measured at the electrochemical workstation CH1660E. X-ray photoelectron spectroscopy was recorded using Kratos AXIS Supra (Shimadzu, Kyoto, Japan).

### 2.4. Electrochemical Test

In this experiment, electrochemical tests were performed using a standard three-electrode system to evaluate the electrochemical hydrogen precipitation performance of the catalyst. The CHI660E electrochemical workstation was used to load the powder samples to be tested onto a glassy carbon electrode of diameter ϕ = 3 mm with 5 wt% concentration of Nafion solution at 3.5 mg cm^−2^. The electrode prepared as described above was used as the working electrode, the C electrode was used as the counter electrode, and Ag/AgCl (saturated KCl-filled) was used as the reference electrode. The LSV curves were obtained by reversible hydrogen electrode potential conversion, and the potential conversion equation for Ag/AgCl was

(7) $$E\left(vs.\;RHE\right)=E\left(vs.\;\mathrm{Ag}/\mathrm{AgCl}\right)+0.0592pH+0.197$$

The electrolyte was a 0.5 mol L^−1^ H_2_SO_4_ solution and linear voltammetry (LSV) was tested at a sweep rate of 5 mV s^−1^. The electrochemical active area (ECSA) of the catalysts was evaluated by testing the double layer capacitance (C_dl_), which was subjected to cyclic voltammetry (CV) at sweep rates of 20 mV s^−1^, 40 mV s^−1^, 60 mV s^−1^, 80 mV s^−1^, and 100 mV s^−1^ at potentials ranging from 0.15 to 0.25 V. The catalysts were tested at a sweep rate of 5 mV s^−1^. The electrochemical impedance (EIS) of the catalyst was measured in the frequency range of 100,000–0.1 Hz to evaluate the electrochemical hydrogen precipitation performance of the catalyst. The stability of the catalysts was evaluated by recording the changes in the LSV curves before and after 1000 cycles of CV testing and recording the curves for 12 h. EIS was fitted with the program Zview and all experimental results are reproducible.

## 3. Results and Discussion

### 3.1. Characterization of Electrocatalysts

**Figure 1:**
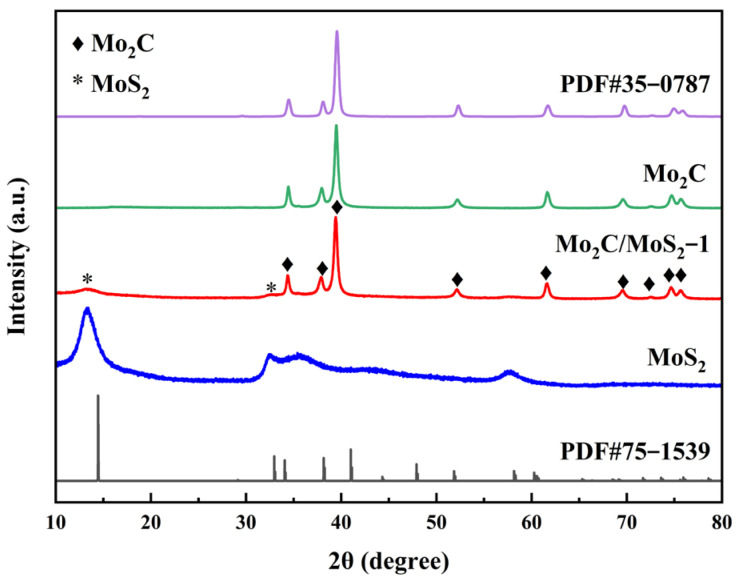
XRD pattern of Mo_2_C, MoS_2_, and Mo_2_C/MoS_2_.

Figure 1 elegantly delineates the X-ray diffraction (XRD) spectra of Mo_2_C; MoS_2_; and their composite, Mo_2_C/MoS_2_, catalysts. Within these spectra, distinct peaks at 34.4°, 37.9°, and 39.5° are observable, corresponding, respectively, to the (100), (002), and (101) lattice planes of β-Mo_2_C crystals. These peaks are congruent with the salient features of the β-Mo_2_C standard PDF card (PDF#35-0708). The MoS_2_ sample, synthesized via hydrothermal methods, exhibited characteristic peaks at 13.5° and 32.4°. These are indicative of the (002) and (100) facets of MoS_2_ crystals, thus confirming the efficacy of the hydrothermal synthesis in crafting MoS_2_. However, it can be observed that the position of the MoS_2_ diffraction peak was low compared to the 2θ value corresponding to the standard peak of 2H-phase MoS_2_, indicating the existence of multiple crystalline phases in the synthesized MoS_2_ samples. 1T-phase MoS_2_ requires lower reaction temperatures and shorter reaction times compared to the 2H-phase MoS_2_, and the position of its (002) crystalline diffraction peaks was around 9°, which resulted in the leftward diffraction peak position. Nevertheless, a diminution in peak intensity is noted in the XRD pattern of the Mo_2_C/MoS_2_ composite due to the 2D layered structure and diminutive grain size of MoS_2_. This nuanced two-dimensional structure manifested less intense diffraction peaks with broader half-peak breadths in pristine MoS_2_ samples. In contrast, β-Mo_2_C displayed pronounced, acutely defined peaks due to enlarged grain size and the formation of nanorods through the high-temperature carbonization of molecular clusters. Consequently, in the composite samples, β-Mo_2_C’s peaks dwarfed those of MoS_2_ in intensity. Yet, vestiges of MoS_2_′s diffraction peaks at 13.5° and 32.4° remained discernible, substantiating the coexistence of dual-phase Mo_2_C and MoS_2_ within the composite. Importantly, the absence of extraneous stray peaks corroborates the sample’s compositional purity.

**Figure 2:**
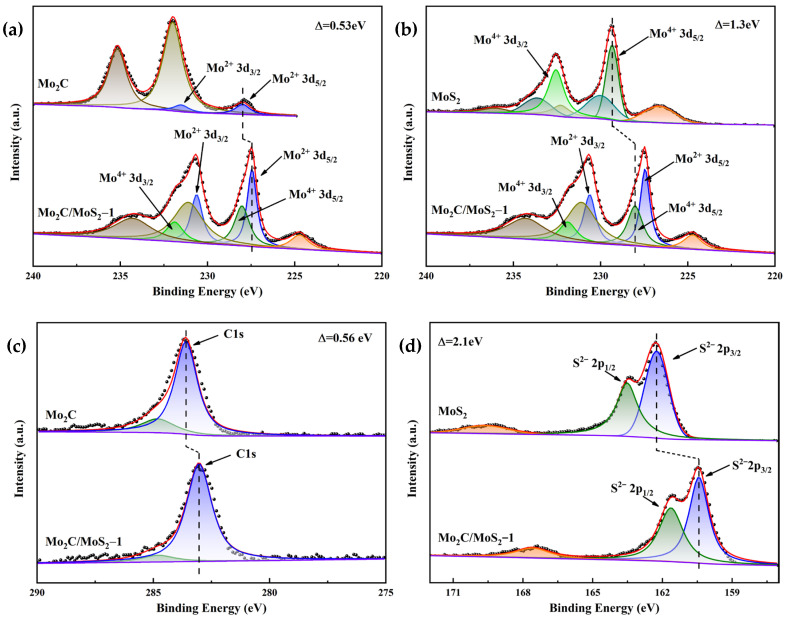
XPS images of Mo_2_C, MoS_2_, and Mo_2_C/MoS_2_-1 composite catalysts: (**a**,**b**) Mo3d; (**c**) C1s; (**d**) S2p.

Analysis of the X-ray photoelectron spectroscopy (XPS) spectra reveals the distinctive peaks at 231.5 eV and 227.9 eV in Figure 2a, which are attributed to the 3d_3/2_ and 3d_5/2_ orbitals of divalent molybdenum (Mo^2+^) in Mo_2_C, respectively. Conversely, peaks observed at 235.2 eV and 231.2 eV correspond to the 3d_3/2_ and 3d_5/2_ orbitals of hexavalent molybdenum (Mo^6+^) in MoO_3_ oxides, signifying oxidative processes affecting the sample surfaces during both the reaction phase and preservation. The Mo_2_C/MoS_2_-1 composite catalysts manifested characteristic peaks at 230.6 eV and 227.4 eV, indicative of the Mo^2+^ 3d_3/2_ and 3d_5/2_ orbitals of the Mo element. These peaks were shifted by 0.53 eV to lower binding energies relative to those of Mo_2_C alone, suggesting an augmented electron cloud density around the composite samples [28]. This phenomenon correlates with a decrease in electrochemical impedance and an enhancement in electrocatalytic performance. Figure 2b corroborates the findings from electrochemical characterization. Peaks at 230.6 eV and 227.4 eV correspond to the presence of tetravalent molybdenum (Mo^4+^) 3d_3/2_ and 3d_5/2_ orbitals characteristic of MoS_2_. In contrast, peaks at 236.0 eV and 232.2 eV are ascribed to MoO_3_, while those at 233.6 eV and 230.0 eV denote an intermediary oxidation state of molybdenum (Mo^ε+^) with 4 < ε < 6. The intermediate oxidation state, as evidenced by the peaks, indicates that the MoS_2_ samples also experienced surface oxidation; notably, the peak at 226.6 eV originated from S2s interference.

In the Mo_2_C/MoS_2_-1 samples, the peaks at 231.8 eV and 228.0 eV are ascribed to MoS_2_ components’ Mo^4+^ 3d_3/2_ and 3d_5/2_ orbitals. They shifted toward lower binding energy relative to the MoS_2_ sample by approximately 1.3 eV. This shift suggests that the electron cloud density around the MoS_2_ component increased upon composite formation, with the Mo_2_C component transferring more free electrons to the MoS_2_ due to Mo_2_C’s role as an electron donor, enriching the electron milieu surrounding MoS_2_. The binding energy discrepancies, denoted by Δ = 3.2 eV for Mo^2+^ spin-split orbitals and Δ = 3.8 eV for Mo^4+^ spin orbitals, differed from those in the Mo_2_C (Δ = 3.6 eV) and MoS_2_ (Δ = 3.2 eV) samples, respectively. These differences indicate a successful heterostructure of the composites, leading to an optimized electronic architecture conducive to superior catalytic performance [29]. The shifting to lower binding energies is also witnessed in the characteristic C1s peaks at 283.0 eV for Mo_2_C/MoS_2_ and 283.6 eV for Mo_2_C, as noted in Figure 2c. Lastly, Figure 2d showcases peaks at 161.6 eV and 160.4 eV for the Mo_2_C/MoS_2_-1 samples, corresponding to the S2^−2^ p_1/2_ and S2^−2^ p_3/2_ orbitals, respectively. These peaks were discernibly shifted towards lower binding energies compared to the 163.5 eV and 162.2 eV peaks of MoS_2_, embodying the changing electron densities in these subsidized structures.

**Figure 3:**
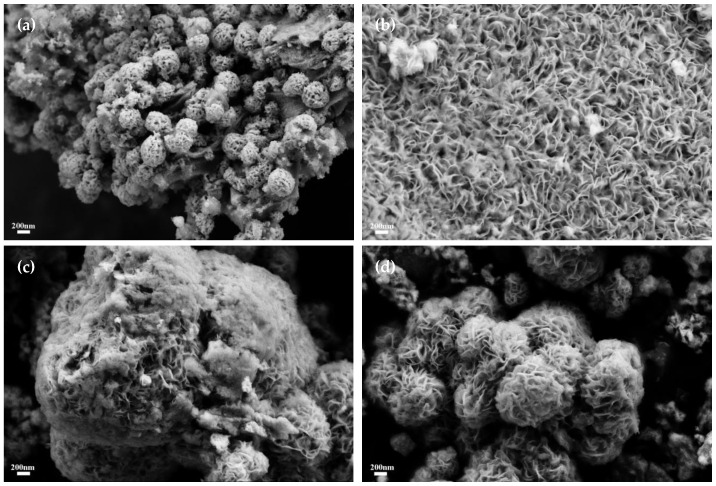
SEM images of catalyst samples: (**a**) Mo_2_C; (**b**) MoS_2_; (**c**,**d**) Mo_2_C/MoS_2_.

The specimens underwent meticulous examination utilizing scanning electron microscopy (SEM), with the corresponding findings delineated in Figure 3. Observational analysis of Figure 3a reveals that molybdenum carbide (Mo_2_C) assumed an elongate ellipsoidal morphology, with each particle approximately 80 nm in diameter, which coalesced to constitute substantial nanorods. These nanorods exhibited a porous exterior, spanning sizes from 400 to 600 nm. Subsequent inspection of Figure 3b unveils a scanning electron micrographic depiction of molybdenum disulfide (MoS_2_), which displays a composition of stratified nanofloral configurations. The distinctly layered petal-like structure corroborates the dimensional stratification intrinsic to MoS_2_. Upon amalgamation of the two entities, as depicted in Figure 3c, the Mo_2_C phase receded nearly beyond detection; nonetheless, one can discern the presence of nanofloral spherical entities, ranging in size from 600 to 800 nm. This indicates a transformative alteration of MoS_2_′s inherent cuboidal morphology post-composite, with the spherical nanofloral architecture potentially unveiling an augmented array of reactive sites. Figure 3d provides a vista into the amalgamated growth of Mo_2_C and MoS_2_ grains through mutual intercalation. Post-combination morphological transformation yielded predominant agglomerations of spherical Mo_2_C particles, the surfaces of which were adorned with the nano-floral constructions of MoS_2_, substantiating a successful synthesis of Mo_2_C/MoS_2_ heterojunction semiconductor catalysts.

**Figure 4:**
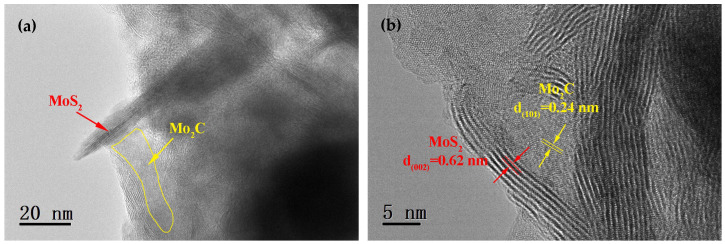
TEM image of Mo_2_C/MoS_2_ composite catalysts: (**a**) Mo_2_C/MoS_2_ composite catalyst; (**b**) localized enlarged view.

In the micrographs obtained via transmission electron microscopy of the Mo_2_C/MoS_2_ heterojunction catalysts, one can discern the characteristic striping of MoS_2_ lamellae interspersed with Mo_2_C crystallites. The MoS_2_ had a predilection for ensheathing the Mo_2_C nuclei, engulfing them in a continuous veil that contributed to the predominant MoS_2_ crystalline façade evident in scanning electron microscopy depictions. A proliferation of defects within the superficial MoS_2_ layers festooning the Mo_2_C granules correlated with an augmented exhibition of dynamic sites, consequently enhancing the catalyst’s overall reactivity. The high-resolution transmission electron microscopy delineation in Figure 4b revealed lattice fringes with an interplanar distance of 0.62 nm, denoting the (002) planes of MoS_2_ crystals, while those fringes with a separation of 0.24 nm are ascribed to the (101) planes of Mo_2_C crystals—a measurement marginally exceeding the canonical value of 0.228 nm. This subtle expansion intimates a tensile distortion within the Mo_2_C matrix as occasioned by the admixture with MoS_2_, a hallmark of the heterostructures’ synthesis through the successful amalgamation of the constituent Mo_2_C and MoS_2_ materials.

### 3.2. Electrochemical Results of Electrocatalysts

**Figure 5:**
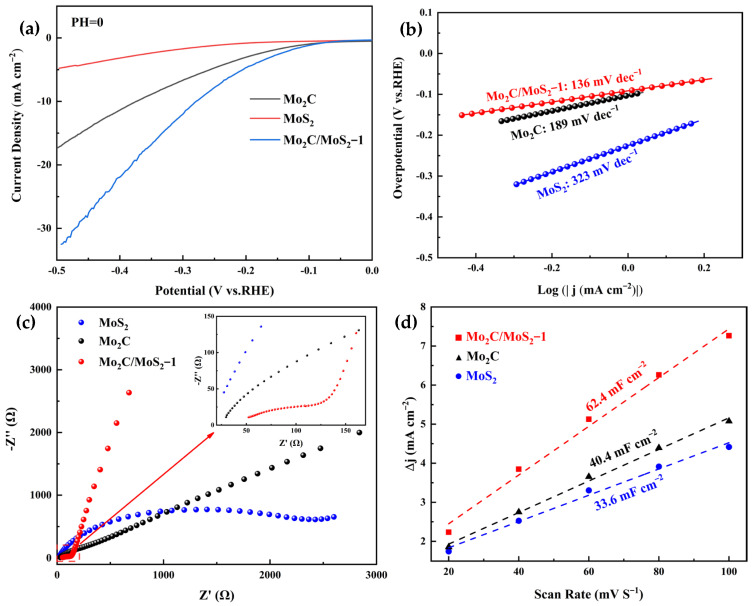
Electrochemical test plots of Mo_2_C, MoS_2_, and Mo_2_C/MoS_2_ composite catalysts: (**a**) LSV; (**b**) Tafel slope; (**c**) EIS; (**d**) ECSA.

Figure 5 depicts the electrochemical evaluation results for the various specimens. As evidenced by Figure 5a,b, the Mo_2_C/MoS_2_ composite catalyst exhibited minimal overpotential at an identical current density. Specifically, this catalyst manifested an overpotential of merely 278 mV at 10 mA cm^−2^ current density, thereby signifying its superior hydrolytic proficiency for electrochemical hydrogen evolution in contrast to the pristine specimen. Concurrently, the Tafel inclination for the Mo_2_C/MoS_2_ composite experienced a considerable descent to 136 mV dec^−1^, markedly lower than the 189 mV dec^−1^ for undiluted Mo_2_C and the 323 mV dec^−1^ for unadulterated MoS_2_. This observation implies that the composite catalyst substantially diminished the activation energy requisite for the hydrolytic process when paralleled with the pure specimen. Furthermore, Figure 5c unveils the electrochemical impedance spectroscopy (EIS) representations for the unmingled Mo_2_C, MoS_2_, and the composite catalyst. The equivalent circuit diagram and specific values are shown in Table 1. It becomes apparent that the composite samples exhibited a reduced arc radius within the high-frequency region than their unmingled counterparts, a factor that intimates a lower impedance and an accelerated rate of electron transference. This enhancement facilitates continuous electron conveyance and chemical reactions, culminating in an augmented electrochemical efficacy for hydrogen evolution.

Due to the direct linear relationship between the ECSA of a catalyst and the magnitude of its C_dl_, the ECSA computation of materials can be inferred by the measurement of their C_dl_. As depicted in Figure 5d, the ECSA of the Mo_2_C/MoS_2_-1 composite catalyst is discerned to be 62.4 mF cm^−2^, which notably surpasses that of the isolated Mo_2_C at 40.4 mF cm^−2^ and MoS_2_ at 33.6 mF cm^−2^. This observation indicates that the composite catalyst acquired an enlarged electrochemically active specific surface area post-synthesis, which resulted in a greater density of active sites. Consequently, this enhancement led to improved electrochemical hydrogen evolution performance, corroborating the outcomes previously illustrated by the polarization curves and Tafel slopes. An excessively high Tafel slope indicates that the Volmer reaction rate is slow, which can limit the reaction rate of the Heyrovsky reaction with the Tafel reaction, further affecting the overall conversion rate of the reaction. The ECSA of the catalyst was significantly increased after the composite of the two, which drastically reduced the Tafel slope of the catalyst and greatly accelerated the rate of electron transfer to produce H* faster. It indicates that the heterogeneous structure can form a high-speed electron transfer channel and dramatically improve the catalytic activity.

**Figure 6:**
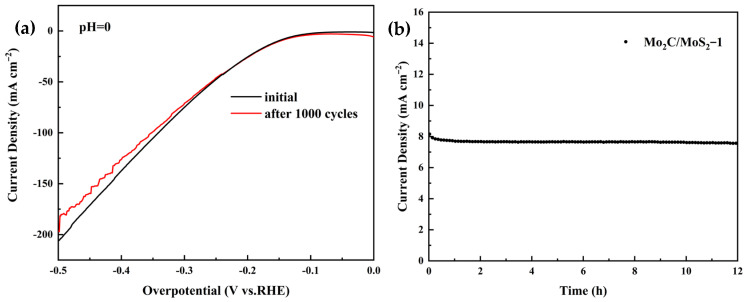
Stability test of Mo_2_C/MoS_2_-1 composite catalyst: (**a**) LSV plot after 1000 cycles of CV; (**b**) its curve plot.

The service stability of the catalysts was tested by cyclic voltammetry and chronoamperometry, and the test results are shown in the following figures: Figure 6a shows the linear voltammetry curves of the Mo_2_C/MoS_2_-1 composite catalysts before and after 1000 cycles at a sweep rate of 100 mV s^−1^, which shows that the overpotential of the catalysts increased slightly after the cycle, with little change compared with that before the cycle. Figure 6b shows the chronoamperometric current curve of the Mo_2_C/MoS_2_-1 composite catalyst with a starting voltage of 297 mV, and the results indicate that the current density was almost unchanged under the test condition of 12 h acidic electrolyte. The above results indicate that the Mo_2_C/MoS_2_-1 composite catalyst exhibited good stability under long-time endurance tests.

### 3.3. Characterization of Composite Catalysts with Different Molar Ratios

**Figure 7:**
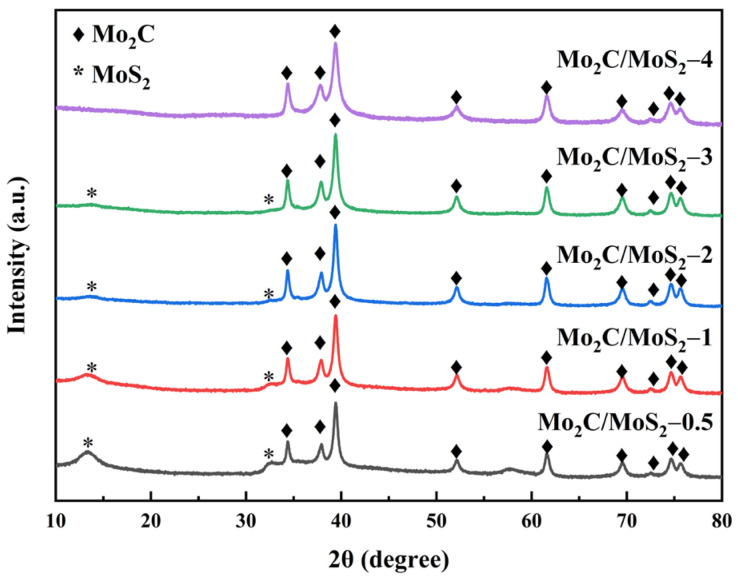
XRD pattern of Mo_2_C/MoS_2_ composite catalysts with different composite ratios.

Figure 7 portrays the X-ray diffraction patterns of heterogeneous catalysts comprised of variable molar proportions of Mo_2_C to MoS_2_. Within these spectrums, one can discern the crystalline signatures of Mo_2_C at ordinal angles (2θ) of 34.4°, 38.0°, 39.4°, 52.1°, 61.5°, 69.6°, 72.4°, 74.6°, and 75.5°. These peaks correspond to the facets of the β-Mo_2_C polymorph (as cataloged in PDF#35-0787) specifically at the (100), (002), (101), (102), (110), (103), (200), (112), and (201) crystallographic planes, respectively, thereby affirming the successful synthesis of Mo_2_C nanoparticles via thermochemical carbonization. Moreover, at diminished Mo_2_C to MoS_2_ ratios, diffraction peaks were discerned at 2θ values of 13.5°, 33.1°, 35.4°, and 57.4°, which are ascribable to the (011), (200), (003), and (110) lattice planes of hexagonal MoS_2_, respectively. The absence of extraneous peaks underscores the purity of the synthesized Mo_2_C/MoS_2_ composited catalysts across the explored molar ratios. As the molar ratio of Mo_2_C to MoS_2_ ascended, a concurrent diminution and broadening of MoS_2_ diffraction peaks were observed, signifying the potential of the composite to attenuate interlayer distances and particle size of the lamellar MoS_2_ structure. This structural alteration renders augmented accessibility to active sites, thereby enhancing the catalytic prowess of the material. Notably, at a molar ratio of 4 units of Mo_2_C to MoS_2_, the peaks representative of MoS_2_ were not detected, attributed to the insufficiency of MoS_2_ presence within the mixture.

**Figure 8:**
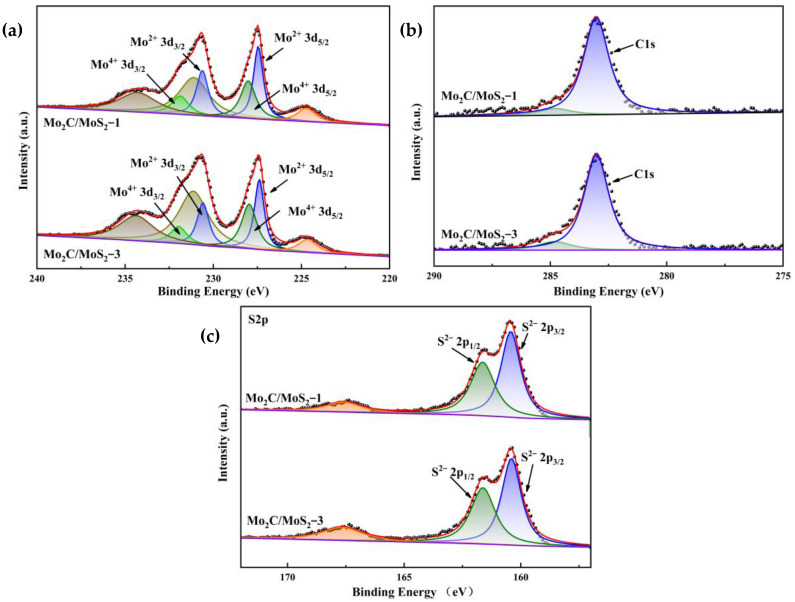
XPS patterns of Mo_2_C/MoS_2_ composite catalysts with different composite ratios: (**a**) Mo3d; (**b**) C1s; (**c**) S2p.

Upon examining the XPS spectra, it is discernible that the characteristic peaks at 230.6 eV and 227.4 eV for the Mo_2_C/MoS_2_-1 specimen, as well as the analogous peaks for the Mo_2_C/MoS_2_-3 specimen depicted in Figure 8a, correspond to the Mo^2+^ 3d_3/2_ and Mo^2+^ 3d_5/2_ states of molybdenum, respectively, signifying the presence of Mo_2_C. Additionally, the peaks observed at 231.9 eV and 228.0 eV for the Mo_2_C/MoS_2_-1 sample, along with the peaks at 232.0 eV and 228.0 eV for the Mo_2_C/MoS_2_-3 sample, are representative of the Mo^4+^ 3d_3/2_ and Mo^2+^ 3d_5/2_ states of molybdenum, ascribed to Mo_2_C. Furthermore, the peaks at 231.9 eV and 228.0 eV about the Mo_2_C/MoS_2_-1 specimen and the peaks at 232.0 eV and 228.0 eV relative to the Mo_2_C/MoS_2_-3 specimen correspond to the Mo^4+^ 3d_3/2_ and Mo^4+^ 3d_5/2_ states, which are indicative of MoS_2_. In Figure 8b, the peak positioned at 283.0 eV for both Mo_2_C/MoS_2_-1 and Mo_2_C/MoS_2_-3 is associated with C1s, denoting Mo_2_C. Additionally, Figure 8c reveals that the characteristic peaks at 161.6 eV and 160.4 eV for the Mo_2_C/MoS_2_-1 sample and the identically characterized peaks for the Mo_2_C/MoS_2_-3 sample correspond to the S^2−^ 2p_1/2_ and S^2−^ 2p_3/2_ states, respectively, indicative of MoS_2_, whereas the peak at 167.6 eV is suggestive of SO_4_^2−^, implicating the partial oxidation of S^2−^ in atmospheric conditions. The aforementioned peaks are consistent across the board with no noticeable shift, implying that modifications in the molar ratio exert negligible influence on the heterojunction between Mo_2_C and MoS_2_. This constancy alludes to the relative stability inherent in the heterostructural composition of the Mo_2_C/MoS_2_ composite catalyst.

**Figure 9:**
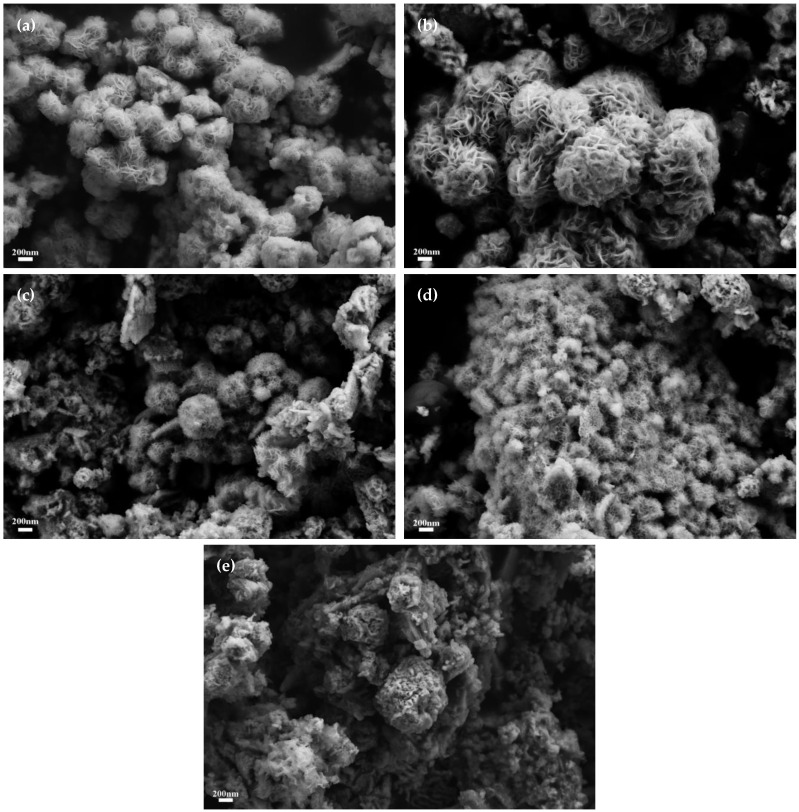
SEM images of Mo_2_C/MoS_2_ composite catalysts with different composite ratios: (**a**) Mo_2_C/MoS_2_-0.5; (**b**) Mo_2_C/MoS_2_-1; (**c**) Mo_2_C/MoS_2_-2; (**d**) Mo_2_C/MoS_2_-3; (**e**) Mo_2_C/MoS_2_-4.

Figure 9 delineates the scanning electron microscopy (SEM) representations of the composite catalysts across varying molybdenum carbide to molybdenum disulfide (Mo_2_C/MoS_2_) molar ratios. Observations reveal a diminution in MoS_2_ crystallite dimensions concomitant with an escalation in Mo_2_C/MoS_2_ molar proportionality. Precisely, the crystallite dimensions approximate 300 nm for Mo_2_C/MoS_2_-0.5; however, this metric contracts to approximately 60–100 nm for the Mo_2_C/MoS_2_-3 specimen, with the reduced crystallite size engendering a commensurately augmented specific surface area. Consequently, this engenders a notably superior electrochemical efficacy in hydrogen evolution reactions.

After the integration of the two constituents, the inherently two-dimensional stratified morphology of MoS_2_ underwent a transition towards a nanoflower spherical configuration, which is indicative of a considerable morphological transfiguration attributed to the influence of Mo_2_C. Owing to the layered architecture, the lion’s share of catalytically active sites within MoS_2_ was predominantly localized at the laminae peripheries. Nonetheless, the emergent nanoflower spherical structure precipitated by the composite formation facilitated the generation of additional active cross-sections. In concert with this morphological evolution, the inherent strata manifested numerous intrinsic strain-induced defects, thereby proliferating the density of active sites conducive to catalytic processes.

### 3.4. Electrochemical Results of Composite Catalysts with Different Molar Ratios

**Figure 10:**
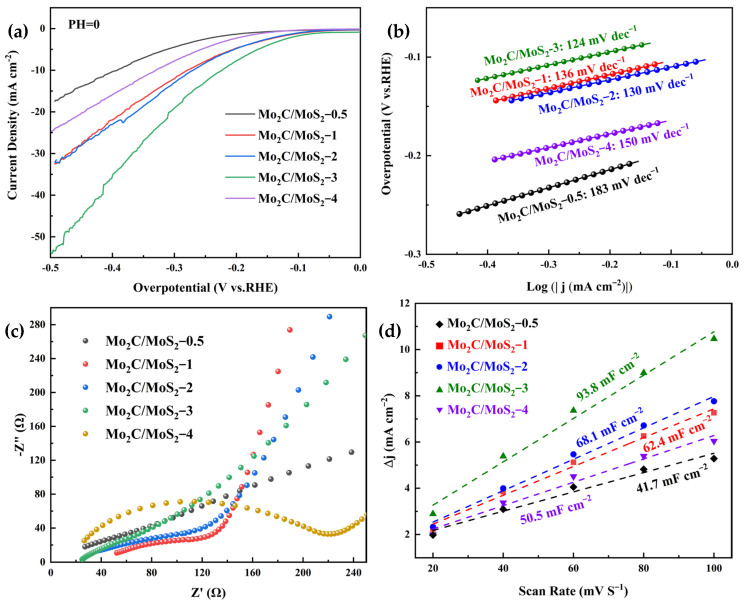
Electrochemical performance of Mo_2_C/MoS_2_ composite catalysts with different composite ratios: (**a**) LSV; (**b**) Tafel slope; (**c**) EIS; (**d**) ECSA.

Figure 10 delineates the electrocatalytic hydrogen evolution prowess of composite catalysts with varying molar ratios of Mo_2_C to MoS_2_, assayed in an aqueous sulfuric acid solution of 0.5 mol L^−1^. As discerned from the linear sweep voltammetry profiles illustrated in Figure 10a, the Mo_2_C/MoS_2_-0.5 composite mandated the highest overpotential for achieving a current density of 10 mA cm^−2^, in stark contrast to the Mo_2_C/MoS_2_-3 composite, which required the least overpotential at the equivalent current density, signifying its superior electrocatalytic hydrogen evolution capability. Corresponding to these findings, Figure 10b presents the electrocatalytic hydrogen evolution attributes of the catalyst variants stratified by their molar ratios, congruent with the linear sweep voltammetry data: an onset overpotential of 183 mV dec^−1^ for Mo_2_C/MoS_2_-0.5 and 150 mV dec^−1^ for Mo_2_C/MoS_2_-4. This suggests that a molar ratio of Mo_2_C to MoS_2_ that is either excessively low or high is detrimental to the electrocatalytic efficacy of the composites. The Mo_2_C/MoS_2_-3 composite exhibited the most negligible Tafel gradient, an indicator that this particular formulation can efficaciously mitigate the activation barrier for the electrocatalytic hydrogen evolution process, hence displaying unparalleled performance. Figure 10c exposes the electrochemical impedance spectroscopy (EIS) profiles for the composites across varying molar ratios, echoing the aforementioned empirical data: the Mo_2_C/MoS_2_-3 composite’s Nyquist plots revealed the least pronounced curvature within the high-frequency electron transport domain, indicative of optimal electrochemical performance. The equivalent circuit diagram and specific values are shown in Table 2.

Upon augmentation of the composite ratio, Mo_2_C, serving as an electron donor, endowed MoS_2_ with an enhanced concentration of electrons, thereby diminishing its electrochemical impedance. The Mo_2_C/MoS_2_-3 samples, with a surplus of readily available free electrons, demonstrated enhanced electrocatalytic activity and were adept at promoting synergistic electron transfer, which accelerates the reduction of protons to hydrogen. Within the array of composite catalysts with varying composite ratios, the ECSA of the catalysts was similarly deduced through the evaluation of the C_dl_ of the materials under investigation. In Figure 10d, the graphical depictions reveal that the Mo_2_C/MoS_2_-3 composite manifested the highest ECSA, registering a capacitance of 93.8 mF cm^−2^. This represents a marked increase of 132% over the pristine Mo_2_C catalyst and an augmentation of 179% in comparison to the unadulterated MoS_2_ catalyst. As the composite ratio of Mo_2_C to MoS_2_ ascended, there was a discernible upward trajectory in the electrochemical active surface area of the composite catalysts. However, the ECSA of the Mo_2_C/MoS_2_-4 sample exhibited a subtle decline, which can be conjectured to result from an overly elevated Mo_2_C to MoS_2_ composite ratio. Such a ratio may diminish the synergetic interaction between the two constituents, predominantly relegating Mo_2_C to serve as the principal component of the catalyst.

Mo_2_C is a narrow-band semiconductor material with a forbidden bandwidth of 1.26 eV, and MoS_2_ has a forbidden bandwidth of 2.31 eV, and a type-II heterostructure will be formed after compositing the two. Due to the difference in electrical conductivity between Mo_2_C and MoS_2_, the Mo_2_C/MoS_2_ composite catalyst will form a heterostructure similar to a P-N junction at the composite interface [30,31]. When the PN junction-type heterojunction is formed, the electrons close to the P-N interface will diffuse to the P region, while the holes close to the P-N interface will diffuse to the N region, leaving the negatively charged P region ions. Due to the high concentration of negatively and positively charged ions assembled at the semiconductor interface, a built-in electric field will be formed, which can drive the electrons and holes to be transported in the opposite direction, greatly enhancing the charge separation efficiency. For HER, the strong interfacial electric field formed by the PN junction-type heterojunction can significantly increase the d-band center of the catalyst, enhance the adsorption of H*, and contribute to the diffusion of metal ions. A comparison of the results of this work with those of other researchers is shown in Table 3

**Figure 11:**
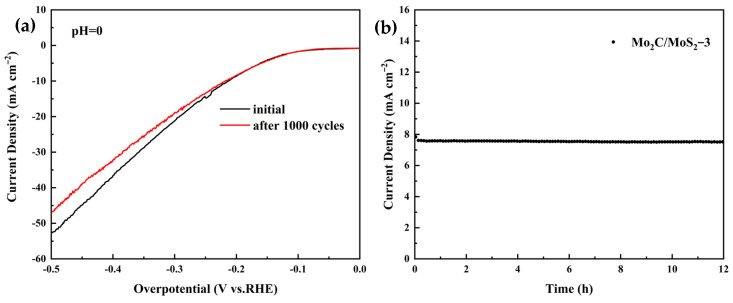
Stability test of Mo_2_C/MoS_2_-3 composite catalyst: (**a**) LSV plot after 1000 cycles of CV; (**b**) its curve plot.

Figure 11 shows the side view of the stability of the Mo_2_C/MoS_2_-3 catalysts. It can be seen from Figure 11a that the cyclic voltammetry test was carried out at a sweep rate of 100 mV s^−1^, and after cycling for 1000 laps, the LSV curves of the Mo_2_C/MoS_2_-3 catalysts showed a relatively small change, and the overpotentials slightly increased at the same current density, which indicated a slight decrease in the catalytic activity of the catalysts. Figure 11b shows that it curve of the Mo_2_C/MoS_2_-3 catalyst showed no obvious fluctuation after 12 h of the timed current test at an initial spot level of 223 mV, which exhibited good stability. The above results indicate that the Mo_2_C/MoS_2_-3 catalysts have excellent stability in long-time electrochemical reactions.

## 4. Conclusions

In summation, Mo_2_C nanoparticles were efficaciously synthesized through a procedure of high-temperature carbonization, subsequently leading to the creation of Mo_2_C/MoS_2_ composite catalysts via the hydrothermal method. Characterization of these composites utilizing advanced material characterization methodologies, such as X-ray diffraction (XRD), scanning electron microscopy (SEM), transmission electron microscopy (TEM), and X-ray photoelectron spectroscopy (XPS), has unequivocally confirmed the successful integration of Mo_2_C into a heterogenous structural amalgamation with MoS_2_. This fusion has substantially curtailed the proclivity of MoS_2_ to aggregate, thereby engendering a prolific increase in defects within the MoS_2_ crystals and unveiling a greater quantity of catalytically active sites. The potential for further enhancement of the composite catalysts’ electronic structure lies in the modulation of the constituent ratios of Mo_2_C to MoS_2_, which precipitates enhanced kinetics in the adsorption, desorption, and reduction processes of H^+^ ions. When juxtaposed with the singular Mo_2_C and MoS_2_ crystals, it is evident that the catalytic efficacy of the composite is markedly superior. Electrochemical assessments, specifically hydrogen evolution reaction (HER) studies, demonstrated that the Mo_2_C/MoS_2_-3 sample heralded an overpotential of merely 224 mV and a Tafel slope of 124 mV dec^−1^ at a current density of 10 mA cm^−2^, showcasing exceptional HER performance. This remarkable performance is ascribable to the heterostructures interlinking the composite components, where Mo_2_C affords electron conduits by its noble metal-like characteristics, thereby ameliorating the electrical conductivity of MoS_2_. Concurrently, MoS_2_’s stratified structure has a wealth of crystal defects and an expansive specific surface area that avails additional active sites germane to the catalyst’s function. The empirical data coalesce to indicate that the intrinsic heterogeneous structure of the Mo_2_C/MoS_2_ composite catalyst acts to significantly bolster its catalytic prowess.

## Figures and Tables

**Table 1:** Equivalent circuit and impedance fit for electrochemical impedance.

Equivalent Circuit	Catalysts	R_S_ (Ω)	R_CT_ (Ω)
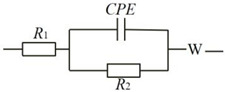	MoS_2_	36.19	890.01
	Mo_2_C	12.21	130.90
	Mo_2_C/MoS_2_-1	44.37	51.86

**Table 2:** EIS values and equivalent circuit diagrams for different composite ratios.

Equivalent Circuit	Catalysts	R_S_ (Ω)	R_CT_ (Ω)
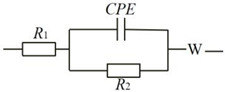	Mo_2_C/MoS_2_-0.5	35.23	70.28
	Mo_2_C/MoS_2_-1	44.37	51.86
	Mo_2_C/MoS_2_-2	39.45	47.86
	Mo_2_C/MoS_2_-3	21.67	39.20
	Mo_2_C/MoS_2_-4	20.25	100.37

**Table 3:** Comparison of the results of this thesis with the experimental results of other researchers.

Catalysts	Over Potential at 10 mA/cm^2^ (mV)	Tafel Slope (mV/dec)	ECSA (mF/cm^2^)	R_CT_ (Ω)	References
Mo_2_C/MoS_2_-3	223.4	124	93.8	39.2	This work
Mo_2_C&MoS_2_@NSC_3_	220	85.5	5.6	/	[32]
Mo_2_C/MoS_2_-25 min	63	53	146	7.5	[33]
NC@Mo_2_C@MoS_2_-(Ni)	205	61.4	4.7	45	[34]
3D MoS_2_@Mo_2_C NS	67	37	64.2	/	[35]

## Data Availability

The data presented in this study are available on request from the corresponding author.
